# Serum and aqueous humor adiponectin levels correlate with diabetic retinopathy development and progression

**DOI:** 10.1371/journal.pone.0259683

**Published:** 2021-11-15

**Authors:** Hyun Seung Yang, Young Je Choi, Hee Yong Han, Hak Su Kim, So Hyun Park, Kyung Sub Lee, Sang Hwa Lim, Doo Jin Heo, Sangkyung Choi

**Affiliations:** 1 Graduate School of Convergence Science and Technology, Seoul National University, Seoul, South Korea; 2 Department of Ophthalmology, Seoul Shinsegae Eye Center, Eui Jung Bu, Gyeonggi-do, South Korea; 3 Department of Ophthalmology, Veterans Health Service Medical Center, Seoul, South Korea; 4 Veterans Medical Research Institute, Veterans Health Service Medical Center, Seoul, South Korea; 5 Department of Endocrinology, Seoul Chuk Hospital, Eui Jung Bu, Gyeonggi-do, South Korea; Medical School, University of Zagreb, CROATIA

## Abstract

**Purpose:**

To compare adiponectin (APN) levels in the serum and aqueous humor (AH) and evaluate their association with the development/progression of diabetic retinopathy (DR).

**Methods:**

Diabetic patients with (group 3; n = 59) and without (group 2; n = 39) DR and age- and sex-matched normal subjects (group 1; n = 35) were compared. Duration of diabetes, body mass index, serum HbA1c, vascular endothelial growth factor (VEGF), APN, pentraxin 3 (PTX3), platelet derived growth factor (PDGF), intercellular adhesion molecule-1 (ICAM-1), and APN were measured and analyzed.

**Results:**

One hundred and thirty-three participants were included. Compared to patients without diabetes, diabetic patients with DR had significantly elevated average serum APN levels (5.99±3.89 μg/ml versus 3.51±1.44 μg/ml, P = 0.002) and average AH APN levels (10.94±11.74 ng/ml versus 3.65±3.33 ng/ml, P<0.001). Serum APN was significantly correlated with AH APN (R = 0.512, P<0.001) and AH VEGF (R = 0.202, P = 0.020). The log serum APN was significantly correlated with intraocular cytokines, including log APN, log VEGF, log ICAM, log leptin, log PTX3, log PDGF, angiopoietin, C-reactive protein, and interleukins (IL)-5 and IL-10 (P<0.001, P = 0.020, P<0.001, P<0.001, P = 0.001, P<0.001, P = 0.008, P = 0.009, P<0.001, and P = 0.046, respectively). Log serum VEGF showed a significant correlation only with log AH VEGF (P = 0.001). Multivariate logistic analysis was performed to evaluate the association of DR progression and cytokine concentrations; log Serum APN and log AH APN showed good correlation with the DR progression in each model.

**Conclusions:**

AH APN levels correlated well with DR development and progression. Serum APN could be a better marker for estimating intraocular cytokines, including both intraocular APN and VEGF concentrations in clinical field, than serum VEGF in DR patients.

## Introduction

Diabetic retinopathy (DR) is a major microvascular complication in patients with diabetes. Risk factors for DR include diabetic control and duration, elevated lipid profiles, race, and levels of inflammatory cytokines in the serum and aqueous humor (AH) [1–5]. Well-known cytokines associated with the development and progression of DR include vascular endothelial growth factor (VEGF), matrix metalloproteinases (MMP), platelet derived growth factor (PDGF), interleukin-6 (IL-6), pentraxin-3 (PTX3), and adhesive molecules, such as ICAM and VCAM. Adiponectin (APN) is a 30-kDa adipocyte-derived vasoactive peptide secreted by fat cells [6–9]. APN is known to affect insulin sensitivity, vascular inflammation, atherosclerosis, endothelial dysfunction, and new vessel formation [10–13].

Hyperglycemia can affect systemic and intraocular levels of APN and other circulatory inflammatory mediators and plays a key role in DR progression and development [12]. Consistently, recent reports also suggest that changes in the most abundant circulating adipokines, such as leptin and APN, which are actively involved in metabolic modulation, may contribute to the development of neovascular eye diseases, including proliferative DR [10]. In addition, APN is implicated in neurodegeneration, inflammation, and neovascularization [14–18]. To better understand the role of APN in the systemic inflammation seen in DR, direct comparisons of the various cytokines, including APN, between serum and AH should be performed. However, previous studies on this topic have shown inconsistent results [5, 7, 19–22]. In addition, DR, as a mild form of chronic inflammation, is believed to be more closely associated with systemic vascular inflammation and innate immunity than other retinal diseases, and to our knowledge, the relationship between DR development/progression and serum and AH APN levels has rarely been studied [23–25].

We believe that APN levels are increased in patients with microangiopathy and chronic inflammation. Hadjadj et al. have reported about the elevated APN concentrations observed in patients with DR and neuropathy and Ouchi et al. have reported that APN acts as an anti-inflammatory plasma protein and modulates the vascular remodeling linked to obesity [4, 23, 24, 26]; therefore, we hypothesized that systemic and local APN levels, reflecting microvascular inflammation, could be a marker of DR and its progression and compared these concentrations to the level of VEGF and other cytokines known to be related to DR development and progression. To this end, we performed several analyses: first, we directly compared levels of cytokines, including APN and VEGF, between serum and AH in patients with DR; second, we analyzed which factors are most significantly related to the progression and development of DR; and third, we assessed the utility of serum APN for predicting the level of various intraocular cytokines in DR patients.

## Methods

### Study design

Participants over 40 years of age who planned to undergo cataract surgery were consecutively recruited from the outpatient clinic of the Division of Ophthalmology at the Ophthalmology Department of Veterans Health Service Medical Center, South Korea, between March 2020 and February 2021. Approval from the ethics committee of the Veterans Health Service Medical Center was obtained (approval number: 2019-12-009-010), and informed consent was obtained from all patients. The study protocol adhered to the tenets of the Declaration of Helsinki. All patients underwent basic physical examinations, blood sampling, and complete eye examinations, which included best-corrected visual acuity, tonometry, bio-microscopy, and ophthalmoscopy using either a 90-diopter lens or indirect ophthalmoscopy following pupil dilation to check for DR grade and retinal abnormality before cataract surgery, followed by a 0.1 ml anterior chamber tap intraoperatively. If a patient had severe cataract preventing grading of DR preoperatively, a retinal exam was conducted one month after cataract surgery. Patients were excluded if they had a history of severe medical problems, including uncontrolled hyperlipidemia, hypertension, cardiac disease, cerebral disease, liver disease, immunological disorders, cancer, severe infection, major surgery, or severe ocular disease. Proliferative DR (PDR) cases were also excluded from this study. Ocular disease was defined as a history of ocular surgery, trauma, corneal degenerative changes affecting visual acuity, uveitis, retinal vascular occlusion, age-related macular degeneration, retinal detachment, glaucoma, central serous chorioretinopathy, ocular tumor, and unknown vasculitis. Additionally, patients with uncontrolled abnormal hepatic function tests (more than two-fold increase over the normal limit), undergoing dialysis, or with abnormal leukocyte counts were also excluded from the present study. After exclusions, a total of 59 diabetes mellitus (DM) patients with DR (group 3), 39 age- and body mass index (BMI)-matched DM patients without DR (group 2), and 35 age- and BMI-matched normal subjects (group 1) were enrolled and underwent all examinations. For subgroup analysis, group 3 was further divided into the following five subgroups: mild non-proliferative diabetic retinopathy (NPDR), moderate NPDR, severe NPDR, PDR, and patients who underwent pan-retinal photocoagulation (PRP), based on fundus examination during screening or just after the surgery. DR was graded as the absence of apparent retinopathy (subgroup 1), while mild NPDR (subgroup 2), moderate NPDR (subgroup 3), and severe NPDR (subgroup 4) were graded based on the ETDRS study [27]. PRP was conducted at least six months before the surgery in subgroup 5. The control group without DM (group 1) was set as subgroup 0. All fundus examinations were performed by two trained ophthalmologists (HYH and YJC) who were blinded to the study design and were graded based on their consensus. In addition, only one eye in each patient was selected using digitalized randomization.

### Laboratory measurements

Venous blood samples were obtained from patients’ forearms after fasting for at least eight hours. Low-density lipoprotein (LDL) cholesterol, high-density lipoprotein (HDL) cholesterol, triglycerides, creatinine, and HbA1c levels were quantified using routine biochemical methods in a certified laboratory at the Department of Laboratory Medicine of Veterans Health Service Medical Center. LDL levels were calculated using the Friedewald formula. A 0.05 cc AH sample was obtained just after side perforation using an MVR blade. Then, an anterior chamber healon injection was injected immediately. Following which, routine cataract surgery was performed. All serum and AH samples were collected and immediately stored at -80°C for subsequent assays. The dilution was 1:2 for the serum and AH, except for the serum APN, which underwent 1:200 dilution after vortexing and centrifuging just before the analysis. The sensitivity thresholds of the cytokines were 148 pg/ml for APN, 9.43 pg/ml for angiopoietin-1, 2.1 pg/ml for VEGF, 116 pg/ml for CRP, 7.67 pg/ml for ESAM, 87.9 pg/ml for ICAM-1, 1.8 pg/ml for IL-2, 9.3 pg/ml for IL-4, 0.5 pg/ml for IL-5, 1.7 pg/ml for IL-6, 1.8 pg/ml for IL-8, 1.6 pg/ml for IL 10, 10.2 pg/ml for leptin, 0.2 pg/ml for PDGF-BB, 39.5 pg/ml for pentraxin 3 (PTX 3), and 238 pg/ml for VCAM-1. Any value below that sensitivity was treated as 0 in the analysis for the average analysis and as a minimum value during logarithm analysis. Serum angiopoietin and serum PDGF-BB levels were not analyzed because of different dilutional requirements. All cytokine concentrations were measured twice (duplication) using a Luminex 200 (Luminex corp., Austin, TX, USA), and the mean values were used in the analysis. The cytokines included angiopoietin-1, ICAM-1, IL-2, IL-4, IL-5, IL-6, IL-8, IL-10, Leptin, Pentraxin 3, VCAM-1, VEGF, PDGF-BB, ESAM, CRP, APN in serum and aqueous humor described above. The concentration values were obtained from the mean fluorescent intensity (MFI) by using Masterplex QT 2010 Software. Standard curves were generated from the reference cytokine gradient concentrations; the concentrations of these cytokines in serum and AH samples were calculated from the standard curves.

### Statistical analysis

Statistical analysis was performed using SPSS Statistics version 17 (SPSS Inc. Chicago, IL, USA) with one-way analysis of variance (ANOVA) in case of parametric analysis; post hoc analysis was performed using Tukey’s test and Bonferroni correction to compare baseline characteristics between the three groups. For non-parametric analysis, Fisher’s exact test was used to compare the three groups. For comparison of two groups, the chi-square test for non-parametric analysis and independent t-test for parametric analysis was used. For trend analysis, Pearson and Spearman correlation coefficients depending on the characteristics of data (normal distribution and scale) and linear-by-linear association method were used. In case, significant scale and dimension differences between two variables and significant outlier in some variables were observed, the Spearman correlation was used. For better assumption of the standard normal distribution, serum and AH ESAM, ICAM-1, VCAM-1, leptin, pentraxin 3, VEGF, and APN measurements were changed into log-transformed values to measure correlations and perform univariate and multivariate analyses between variables. A test for linear trend was calculated based on the average levels of APN in the serum and AH within each group and subgroup. Statistical significance was set at P < 0.05.

Coefficient of variation (CV) was determined by the standard deviation value divided by the average value multiplied by 100. Intra-assay precision was determined for identical samples by assessing each sample a minimum of six times. Inter-assay precision was determined for assaying aliquots of each sample in four to six independent analytical runs.

## Results

### Baseline characteristics

**Fig 1:**
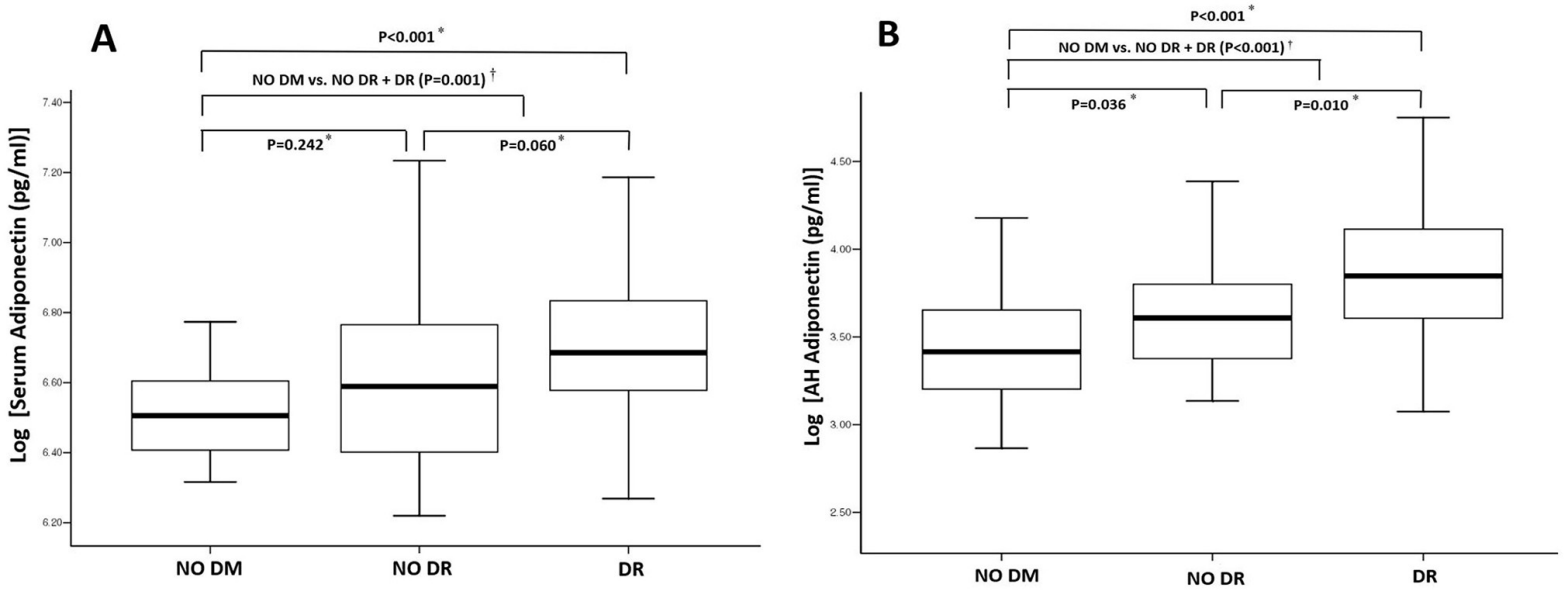
The concentrations of A) log-transformed serum adiponectin and B) log-transformed aqueous humor (AH) adiponectin in the presence of diabetic retinal complication. Thick lines in the middle of the boxes represent the median value for each group. DM, diabetes mellitus; DR, diabetic retinopathy. * One-way analysis of variance (ANOVA) test with post hoc analysis using Tukey’s test and Bonferroni correction. ^†^ Independent T test.

The demographic, clinical, and laboratory characteristics of the 133 participants included in the study are summarized in Table 1. The serum APN concentrations (log APN in serum) for group 1 (n = 35), group 2 (n = 39), and group 3 (n = 59) were 3.51±14.21 μg/ml (6.52±0.15), 4.803.23 μg/ml (6.61 ± 0.25) and 5.99±3.89 μg/ml (6.71 ± 0.23), respectively (Fig 1A, P<0.001). The AH APN concentrations (log APN in AH) for groups 1, 2, and 3 were 3.65±3.33 ng/ml (3.43±0.35), 5.99±5.60 ng/ml (6.61 ± 0.25) and 10935.57±11736.51 pg/ml (6.71 ± 0.23), respectively (Fig 1B, P<0.001). The direct comparison in patients without and with DR using independent T test showed significant difference in both of log Serum APN (p = 0.034) and AH APN (p = 0.004). In addition, the significant concentration difference was observed in several cytokines such as log AH ICAM-1 (p = 0.007), AH IL-5 (p = 0.002), AH PDGF (p = 0.048) and AH VEGF (p = 0.002) in patents with and without DR. One hundred and five (78.9%) participants were male and 28 (21.1%) were female, with a mean age of 75.2 years. Sex, age, BMI, axial length, visual acuity, and lipid profiles were not significantly different among the three groups (*P* = 0.968, *P* = 0.612, *P* = 0.037; no significant difference with Bonferroni correction, *P* = 0.627, and *P* ≥ 0.091, respectively). However, the duration of DM, presence of HTN, statin use, and HbA1c were significantly different among the three groups (*P* < 0.001, *P* < 0.001, *P* = 0.001, and *P* < 0.001, respectively). In group 3, there were 23, 20, 4, and 12 patients with mild NPDR, moderate NPDR, severe NPDR, and PRP, respectively.

**Table 1:** Demographic data and values of various parameters (mean ± standard deviation) between the three groups (Group 1, age- and BMI-matched normal healthy subjects; Group 2, age- and BMI-matched diabetics without DR; Group 3, age- and BMI-matched diabetics with DR).

	Group 1 (n = 35)	Group 2 (n = 39)	Group 3 (n = 59)	*P*-value
Sex (Male:Female)	28:7	31:8	36:13	0.968*
Age (Years)	75.0 ± 6.2	74.6 ± 5.7	75.7 ± 4.1	0.612^†^
BMI, kg/m^2^	25.6 ± 3.4	25.1 ± 3.4	24.9 ± 3.3	0.630^†^
Presence of HTN (Numbers)	19 (54.3%)	33 (84.6%)	52 (88.1%)	<0.001‡
Duration of DM (Years)	0	11.33 ± 7.6	17.71 ± 10.43	<0.001^†^
Axial length (mm)	23.97 ± 1.56	24.32 ± 1.22	23.64 ± 1.13	0.037^†^
ACD (mm)	3.12 ± 0.32	3.18 ± 0.35	3.03 ± 0.42	0.141^†^
Visual acuity (logMAR)	0.10 ± 0.26	0.16 ± 0.36	0.12 ± 0.22	0.627^†^
IOP (mmHg)	14.8 ± 2.8	15.0 ± 3.6	14.8 ± 2.1	0.952^†^
HbA1c (%)	5.80 ± 0.53	6.83 ± 1.04	7.57 ± 1.16	<0.001^†^
Serum Albumin (g/dL)	4.17 ± 0.28	4.14 ± 0.35	4.18 ± 0.37	0.824^†^
Serum HDL (mg/dL)	48.37 ± 12.25	46.46 ± 12.73	42.81 ± 12.22	0.091^†^
Serum LDL (mg/dL)	103.97 ± 32.43	111.41 ± 30.98	107.90 ± 26.88	0.561^†^
Serum TG (mg/dL)	142.77 ± 44.20	149.28 ± 114.90	153.68 ± 108.26	0.873^†^
Statin use (n)	10 (28.6%)	21 (53.8%)	38 (64.4%)	0.003‡
Serum CRP (mg/L)	1.65 ± 2.64	2.03 ± 4.77	2.09 ± 5.32	0.894^†^
Serum BUN (mg/dL)	17.60 ± 5.03	20.43 ± 9.49	23.27 ± 16.16	0.094^†^
Serum Creatinine (mg/dL)	0.96 ± 0.24	0.98 ± 0.29	1.60 ± 1.67	0.008^†^
Serum AST (U/L)	24.94 ± 13.75	28.82 ± 24.20	20.61 ± 6.86	0.039^†^
Serum ALT (U/L)	22.94 ± 16.22	25.46 ± 16.95	21.90 ± 10.21	0.471^†^

ACD, anterior chamber depth; ALT, alanine aminotransferase; AST, aspartate aminotransferase; BMI, body mass index; BUN, blood urea nitrogen; CRP, C-reactive protein; DM, diabetes mellitus; DR, diabetic retinopathy; HDL, high-density lipoprotein; HTN, hypertension; IOP, intraocular pressure; LDL, low-density lipoprotein; TG, triglyceride

* Chi-square test;

^†^ one-way analysis of variance (ANOVA) test;

^‡^ Willis test.

### Differences in serum and AH cytokines among groups (no DM, no DR, and DR)

Serum and AH cytokine levels for the three groups are summarized in Table 2. Log-transformed serum and AH APN showed significant differences among the three groups (P < 0.001 and P < 0.001, respectively). The log AH VEGF showed a significant difference between the three groups (P < 0.001), but serum VEGF did not (P = 0.520). On trend analysis, only serum IL-2, IL-6, and log serum APN levels showed an increasing trend for serum cytokines (all P < 0.001). For AH cytokines, log AH APN, ICAM 1, IL-2, IL-5, IL-6, IL-8, IL-10, log AH leptin, log AH PDGF, log AH PTX 3, and log AH VEGF were significantly correlated with DR development and progression (p < 0.001, p < 0.001, p = 0.027, p < 0.001, p = 0.003, p < 0.001, p = 0.010, p < 0.001, p < 0.001, p = 0.29, and p < 0.001, respectively). Fig 1 also shows a positive correlation between log serum APN and log AH APN with DM and DR development (R = 0.360, p < 0.001 and R = 0.447, p < 0.001, respectively).

**Table 2:** Serum and aqueous humor values of various parameters (mean ± standard deviation) between the three groups (Group 1, age- and BMI-matched normal healthy subjects; Group 2, age- and BMI-matched diabetics without diabetic retinopathy; Group 3, age- and BMI-matched diabetics with diabetic retinopathy).

	Group 1 (n = 35)	Group 2 (n = 39)	Group 3 (n = 59)	*P*-value*
Serum ESAM [pg/ml]	3216.48 ± 1607.36	2650.18 ± 1148.43	3745.86 ± 4272.50	
Log Serum ESAM [pg/ml]	3.47 ± 0.18	3.38 ± 0.20	0.32 ± 0.33	0.371
Serum ICAM-1 [pg/ml]	393932.47 ± 321114.54	523476.66 ± 419730.13	446805.97 ± 344470.10	
Log Serum ICAM-1 [pg/ml]	5.40 ± 0.67	5.53 ± 0.67	5.49 ± 0.56	0.683
Serum IL-10 [pg/ml]	0.99 ± 0.49	1.42 ± 1.59	1.65 ± 3.18	0.405
Serum IL-2 [pg/ml]	0.00 ± 0.00	0.40 ± 1.34	46.62 ± 338.72	0.503
Serum IL-4 [pg/ml]	10.35 ± 19.59	18.04 ± 29.95	47.95 ± 306.78	0.639
Serum IL-5 [pg/ml]f	8.47 ± 4.94	9.06 ± 9.29	9.32 ± 7.73	0.871
Serum IL-6 [pg/ml]	4.12 ± 0.77	4.27 ± 5.67	13.55 ± 75.20	0.569
Serum IL-8 [pg/ml]	8.84 ± 2.66	13.63 ± 20.44	17.05 ± 56.46	0.620
Serum leptin [pg/ml]	9710.90 ± 9910.28	7525.44 ± 7316.43	72372.98 ± 496855.74	
Log Serum leptin [pg/ml]	3.79 ± 0.43	3.72 ± 0.38	0.38 ± 0.50	0.667
Serum pentraxin 3 [pg/ml]	476.42 ± 558.60	421.76 ± 424.19	595.41 ± 949.78	0.489
Log Serum pentraxin 3 [pg/ml]	2.34 ± 0.60	2.36 ± 0.57	2.45 ± 0.53	0.581
Serum VCAM-1 [pg/ml]	1245045.22 ± 362277.25	11895304.46 ± 551987.25	1308450.15 ± 578984.90	
Log Serum VCAM-1 [pg/ml]	6.08 ± 0.12	6.04 ± 0.18	6.08 ± 0.18	0.438
Serum VEGF [pg/ml]	68.17 ± 45.76	66.93 ± 46.47	81.00 ± 70.81	
Log Serum VEGF [pg/ml]	1.70 ± 0.41	1.70 ± 0.36	1.77 ± 0.38	0.520
Serum adiponectin [pg/ml]	3510333.87 ± 1420609.71	4799372.74 ± 3229336.41	5992967.27 ± 3894709.16	
Log Serum adiponectin [pg/ml]	6.52 ± 0.15	6.61 ± 0.25	6.71 ± 0.23	<0.001
aqueous humor Adiponectin [pg/ml]	3654.19 ± 3326.18	5991.75 ± 5559.70	10935.57 ± 11736.51	
Log aqueous humor Adiponectin [pg/ml]	3.43 ± 0.35	3.64 ± 0.33	3.86 ± 0.39	<0.001
aqueous humor Angiopoietin-1 [pg/ml]	19.53 ± 25.77	29.82 ± 71.96	13.51 ± 15.87	0.180
aqueous humor CRP [pg/ml]	6025.67 ± 11608.5	7135.20 ± 18247.30	6153.81 ± 17385.34	0.946
aqueous humor ESAM [pg/ml]	178.25 ± 82.17	220.02 ± 176.01	201.74 ± 163.63	
Log aqueous humor ESAM [pg/ml]	2.19 ± 0.28	2.27 ± 0.21	2.24 ± 0.21	0.309
aqueous humor ICAM-1/CD54 [pg/ml]	1633.55 ± 981.76	1709.50 ± 2466.46	1081.10 ± 1645.22	
Log aqueous humor ICAM-1 [pg/ml]	3.10 ± 0.41	2.97 ± 0.53	2.63 ± 0.64	<0.001
aqueous humor IL10 [pg/ml]	0.00 ± 0.00	0.80 ± 2.42	2.71 ± 9.33	0.108
aqueous humor IL2 [pg/ml]	2.42 ± 3.37	5.11 ± 8.94	5.57 ± 8.49	0.141
aqueous humor IL4 [pg/ml]	1.80 ± 5.09	4.19 ± 26.19	3.97 ± 19.28	0.839
aqueous humor IL5 [pg/ml]	0.96 ± 1.77	2.74 ± 2.71	4.48 ± 2.70	<0.001
aqueous humor IL6 [pg/ml]	44.50 ± 226.57	14.94 ± 49.57	32.09 ± 165.75	0.733
aqueous humor IL8 [pg/ml]	9.07 ± 16.12	12.28 ± 11.51	11.70 ± 7.42	0.436
aqueous humor leptin [pg/ml]	468.25 ± 33.00	477.14 ± 36.36	644.19 ± 1125.01	
Log aqueous humor leptin [pg/ml]	2.67 ± 0.03	2.68 ± 0.03	2.72 ± 0.17	<0.001
aqueous humor PDGF-BB [pg/ml]	2.46 ± 1.66	3.42 ± 1.69	3.85 ± 1.23	<0.001
Log aqueous humor PDGF-BB [pg/ml]	0.24 ± 0.42	0.43 ± 0.40	0.55 ± 0.21	
aqueous humor pentraxin 3 [pg/ml]	198.88 ± 124.55	187.78 ± 138.91	344.87 ± 812.34	
Log aqueous humor pentraxin 3 [pg/ml]	2.23 ± 0.28	2.16 ± 0.33	2.08 ± 0.57	0.408
aqueous humor VCAM-1 [pg/ml]	20911.73 ± 15258.87	27152.30 ± 25104.30	23398.62 ± 19202.68	
Log aqueous humor VCAM-1 [pg/ml]	4.21 ± 0.39	4.32 ± 0.29	4.31 ± 0.21	0.194
aqueous humor VEGF [pg/ml]	61.47 ± 29.94	69.11 ± 35.57	91.18 ± 37.35	
Log aqueous humor VEGF [pg/ml]	1.72 ± 0.30	1.79 ± 0.21	1.92 ± 0.19	<0.001

CRP, C-reactive protein; ESAM, endothelial-cell selective adhesion; ICAM, intercellular adhesion molecule; IL, interleukin; PDGF, platelet-derived growth factor; PTX, pentraxin; VEGF, vascular endothelial growth factor

* One-way analysis of variance (ANOVA) test.

### Relationship between serum and corresponding AH cytokine concentrations

**Fig 2:**
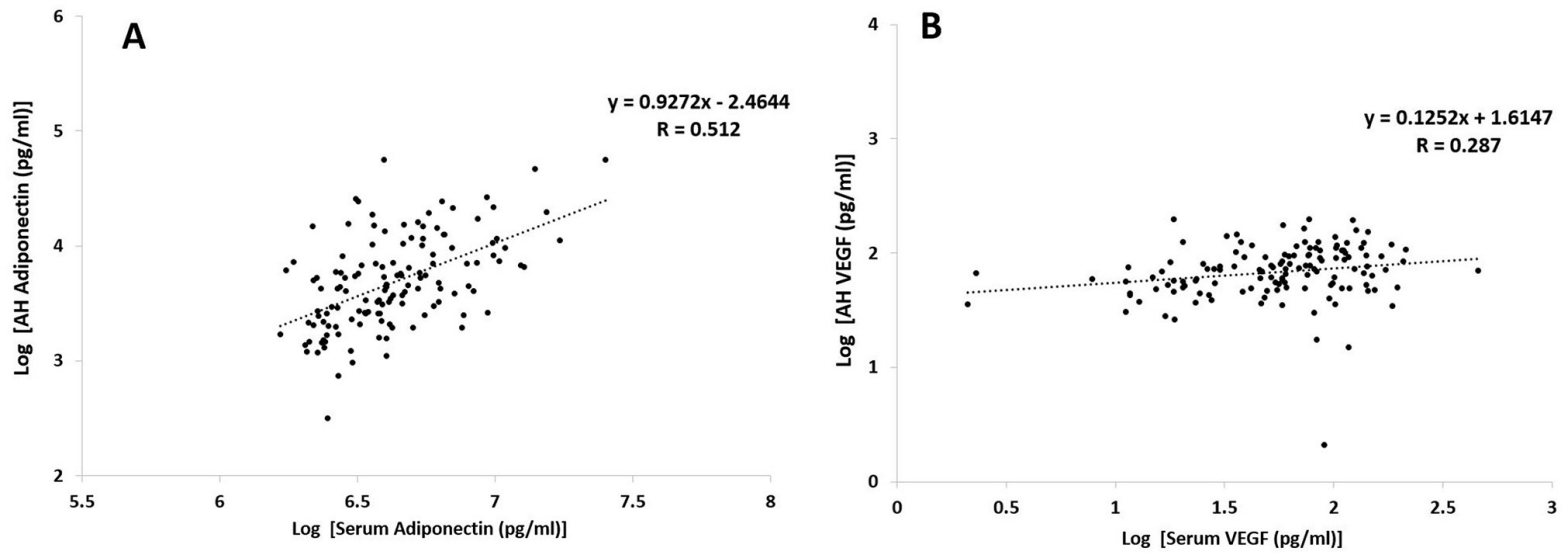
Spearman correlation test between log-transformed A) serum and aqueous humor (AH) adiponectin and B) serum and AH vascular endothelial growth factor (VEGF) in normal and type 2 diabetic patients.

Serum and AH APN and VEGF concentrations showed significant correlation between log serum APN and log AH APN as well as between log serum VEGF and log AH VEGF (Fig 2A; P < 0.001 and Fig 2B; P = 0.001, respectively). In addition, log ESAM, serum IL-2, log IL-6, and leptin concentrations also showed significant correlations with the corresponding cytokine levels (P < 0.001, P = 0.032, P = 0.011, and P = 0.003, respectively). However, there were no significant correlations between serum and the corresponding AH levels of log ICAM-1, log VCAM-1, IL-4, IL-5, IL-8, IL-10, and log PTX3 (all P > 0.05).

Table 3 shows the correlation between serum APN and VEGF levels and AH cytokines. The log serum APN was significantly correlated with log APN, log VEGF, log ICAM, log leptin, log PTX3, log PDGF, angiopoietin, CRP, IL-5, and IL-10 in AH (P < 0.001, P = 0.020, P < 0.001, P < 0.001, P = 0.001, P < 0.001, P = 0.008, P = 0.009, P < 0.001, and P = 0.046, respectively). However, serum VEGF showed a significant correlation with AH VEGF only.

**Table 3:** Correlation using Spearman correlation test between serum adiponectin (APN) and Vascular Endothelial Growth Factor (VEGF) and other intraocular cytokines in Aqueous Humor (AH).

(AH)	Log APN	Log VEGF	Log	Log	Log	Log	Log	Log	Angiopoietin	CRP	IL-2	IL-4	IL-5	IL-6	IL-8	IL-10
(Serum)			ESAM	ICAM-1	VCAM-1	leptin	PTX3	PDGF								
Log APN																
R	0.512	0.202	-0.095	-0.509	0.021	0.370	-0.295	0.370	-0.228	-0.225	0.138	-0.147	0.574	0.155	0.096	0.174
p-value	<0.001	0.020	0.276	<0.001	0.812	<0.001	0.001	<0.001	0.008	0.009	0.112	0.090	<0.001	0.075	0.273	0.046
Log VEGF																
R	0.024	0.287	0.003	0.007	0.020	-0.045	-0.002	-0.045	-0.069	0.070	-0.127	-0.142	-0.056	0.147	0.156	-0.051
p-value	0.783	0.001	0.971	0.940	0.818	0.610	0.981	0.610	0.430	0.425	0.146	0.102	0.519	0.092	0.073	0.558

CRP, C-reactive protein; ESAM, endothelial-cell selective adhesion; ICAM, intercellular adhesion molecule; IL, interleukin; PDGF, platelet-derived growth factor; PTX, pentraxin; VEGF, vascular endothelial growth factor.

### Clinical factors, serum cytokines, and AH cytokines, including APN, as biomarkers for predicting DR progression and development

**Fig 3:**
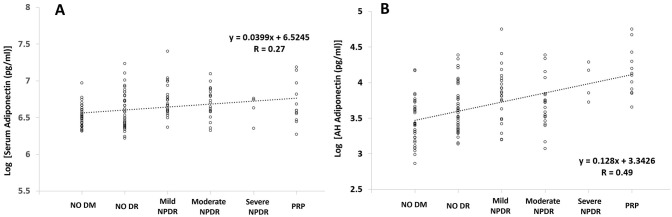
Distributions and trends for log-transformed concentrations using Spearman correlation test of A) serum and 2) aqueous humor (AH) adiponectin in each subgroup of diabetic retinopathy. DM, diabetes mellitus; DR, diabetic retinopathy; NPDR, non-proliferative diabetic retinopathy; PRP, pan-retinal photocoagulation.

Regarding the relationship between the three groups and their clinical characteristics, the duration of DM, presence of HTN, HbA1c, HDL, statin use, and creatinine level were significantly associated with DR progression and development in univariate analysis (P < 0.001, P < 0.001, P < 0.001, P = 0.014, P = 0.001, and P = 0.005, respectively). For the univariate analysis of serum and AH cytokines, the log-transformed serum APN IL-2 and IL-6, and AH APN, IL-2, IL-5, IL-6, IL-8, log PDGF, and VEGF showed a good correlation with DR progression and development (all P ≤ 0.027). However, in multivariate logistic analysis, DR progression and development were only significantly correlated with HbA1c (P = 0.023), log AH APN (P = 0.012), log AH VEGF (P = 0.006), and log AH ICAM-1 (P = 0.022), but not with log AH VCAM-1 (P = 0.127) (Table 4). Fig 3A and 3B also showed an increasing tendency of serum and AH APN levels following DR development and progression in the subgroup analysis (all P < 0.001).

**Table 4:** Multivariate logistic analysis with to determine clinical factors, including cytokines in serum and Aqueous Humor (AH), for estimating the progression between patients without Diabetic Retinopathy (DR) and with DR.

Model 1 (with log Serum APN concentration)				Model 2 (with log AH APN concentration)			
	P value	ORs	95% CI		P value	ORs	95% CI
Log Serum APN, pg/mL	0.046	7.46	1.03–53.91	Log AH APN, pg/mL	0.012	6.25	0.01–7.90
Log Serum VEGF, pg/mL	0.597	1.398	0.40–4.85	Log AH VEGF, pg/mL	0.006	7.433	2.77–508.29
Log AH VEGF, pg/mL	0.010	25.53	2.14–304.50	Log AH ICAM 1 pg/mL	0.022	5.225	0.16–0.87
HbA1c (%)	0.06	1.98	1.21–3.2	Log AH VCAM 1 pg/mL	0.127	2.33	0.017–1.658
				HbA1c (%)	0.023	5.144	1.080–2.863
Hosmer-Lemeshow test	0.885			Hosmer-Lemeshow test	0.889		

APN, adiponectin; VEGF, vascular endothelial growth factor.

### Serum APN as a possible biomarker for predicting AH APN and AH VEGF and its comparison with serum VEGF

Table 5 shows the relationship between serum APN and VEGF levels. The log serum APN showed a relatively good correlation with log AH VEGF (R = 0.202) and log AH APN (R = 0.512). However, log serum VEGF showed a significant correlation with log AH VEGF, but not with log AH APN. There was a relatively weak correlation between log serum APN and VEGF (R = -0.167; P = 0.054); however, the relationship between log AH APN and AH VEGF revealed a relatively good correlation (R = 0.394; P < 0.001).

**Table 5:** Correlation using Spearman correlation test between adiponectin (APN) and Vascular Endothelial Growth Factor (VEGF) in serum and Aqueous Humor (AH).

	Log (serum APN)	Log (serum VEGF)	Log (AH APN)	Log (AH VEGF)
Log (serum APN)	-	R = -0.167 (p = 0.054)	R = 0.512 (p < 0.001)	R = 0.202 (p = 0.020)
Log (serum VEGF)	-	-	R = 0.024 (p = 0.783)	R = 0.287 (p = 0.001)
Log (AH APN)	-	-	-	R = 0.394 (p < 0.001)
Log (AH VEGF)	-	-	-	-

### Intra- and inter-assay precision

Table 6 shows good reproducibility of the intra- and inter-assay precisions for all the assays using quality controls that contain different concentration levels of APN in serum and AH. The Intra-assays of 15 analytes for angiopoietin-1, ICAM-1, IL-2, IL-4, IL-5, IL-6, IL-8, IL-10, Leptin, Pentraxin 3, VCAM-1, VEGF, PDGF-BB, ESAM, and CRP in AH were 5.7%, 2.9%, 4.5%, 5.4%, 13.9%, 2.3%, 2.7%, 4.7%, 7.4%, 18.8%, 4.8%, 5.2%, 2.0%, 5.0% and 12.1%, respectively. Inter-assays of 15 analytes for angiopoietin-1, ICAM-1, IL-2, IL-4, IL-5, IL-6, IL-8, IL-10, Leptin, Pentraxin 3, VCAM-1, VEGF, ESAM, and CRP in AH were 6.1%, 3.3%, 5.4%, 6.2%, 14.9%, 3.5%, 4.3%, 6.5%, 8.9%, 23.9%, 5.2%, 6.3%, 3.1%, 6.7%, and 14.2%, respectively. The Intra-assays of 13 analytes for ICAM-1, IL-2, IL-4, IL-5, IL-6, IL-8, IL-10, Leptin, Pentraxin 3, VCAM-1, VEGF, ESAM, and CRP in serum were 2.1%, 3.8%, 6.7%, 11.5%, 1.9%, 3.2%, 3.9%, 4.8%, 13.0%, 3.7%, 4.1%, 2.8%, and 8.0%, respectively. Inter-assays of 13 analytes for ICAM-1, IL-2, IL-4, IL-5, IL-6, IL-8, IL-10, Leptin, Pentraxin 3, VCAM-1, VEGF, ESAM, and CRP in serum were 3.5%, 4.2%, 8.1%, 15.0%, 3.6%, 4.2%, 4.9%, 6.0%, 15.9%, 5.2%, 5.7%, 5.7%, and 11.5%, respectively.

**Table 6:** Adiponectin (APN) assay in serum and Aqueous Humor (AH).

	Intra -assay precision				Inter -assay precision			
Assay	Expected Nominal (pg/ml)	MFI	Calculated Mean (pg/ml)	%CV	Expected Nominal (pg/ml)	MFI	Calculated Mean (pg/ml)	%CV
Luminex multiplex for serum	216755.0	7342.3	216932.8	4.1	215500.0	8283.1	217021.5	6.4
	72251.7	4600.8	72346.1	3.2	71833.3	5582.7	72317.4	5.3
	24083.9	2196.8	24004.7	2.0	23944.4	2823.8	24042.7	4.0
	8028.0	897.1	8096.6	2.7	7981.5	1167.2	8060.7	4.1
	2676.0	333.1	2652.1	2.5	2660.5	442.3	2660.8	4.0
	892.0	118.9	894.0	3,9	886.8	156.0	895.4	6.7
Luminex multiplex for AH	216755.0	9257.1	216929.5	3.2	215500.0	9443.8	217039.2	6.4
	72251.7	5530.5	72245.2	2.6	71833.3	6240.8	72210.6	4.2
	24083.9	2616.9	24098.8	1.3	23944.4	3076.2	24137.7	3.3
	8028.0	1039.8	8020.1	1.2	7981.5	1233.7	7988.9	2.9
	2676.0	381.9	2679.6	1.6	2660.5	459.2	2693.6	3.4
	892.0	147.9	891.3	5.0	886.8	166.3	888.6	6.8

CV, Coefficient of variation; MFI, Median Fluorescent Intensity.

## Discussion

Adipocytes are not only an important caloric reservoir but also one of the main cells that can produce proactive cytokines that play a critical role in maintaining metabolic homeostasis in the human body [26]. APN is associated with insulin resistance in a wide range of target tissues [11]. Yamauchi et al. demonstrated that administration of APN increases the oxidation of fatty acids in skeletal muscle and suppresses lipid metabolism and lipid concentration in hepatocytes by activating adenosine monophosphate [28]. APN can suppress the expression of glucose-6-phosphatase and phosphoenolpyruvate carboxykinase, thereby suppressing glucose production in the liver [29]. APN also affects energy control in the body by preventing glucose anabolism within hepatocytes and improving fatty acid degradation in myocytes [29]. Insulin resistance is related to an increased risk of microvascular complications, such as DR and nephropathy [23].

By suppressing insulin resistance, APN controls the inflammatory cascade. By preventing apoptosis and inflammation, APN has a positive role in protecting the systemic circulation system and major organs, including the heart, lungs, and colon [30]. The adaptor protein binds directly to intracellular AipoR1 and R2 and controls signal transduction. AdipoR1 and adipoR2 have inherent ceramidase activity [31] and can cause a decrease in intracellular ceramide, a type of sphingolipid [31]. This sphingolipid is believed to play a role in controlling inflammation, sclerotic changes within vessels, and insulin resistance [30, 32].

By controlling inflammation, APN may play a positive role in preventing diabetic microvascular complications. Jung et al. reported that the levels of APN were significantly different according to the presence of each microangiopathy, including retinopathy and nephropathy [24]. Supporting this positive role of APN in DR, Liao et al. reported that the serum APN level and genetic variations in APN receptors were associated with DR (odds ratio (OR) = 1.63, 95% confidence interval (CI) = 1.19–2.25) [4]. In their study of Latino patients with type 2 diabetes, Kuo et al. reported that serum APN is elevated in DR, is positively correlated with DR severity, and maintains a relationship with insulin sensitivity in patients with and without DR [12]. Huang et al. reported limited evidence for the causal role of serum APN in DR risk among Taiwanese patients with diabetes [19]. In addition, APN gene polymorphisms are associated with retinopathy in patients with diabetes [20]. Omae et al. [21] showed that serum APN may be associated with increased retinal blood flow, probably via increased blood velocity and dilated vessel diameter in men with type-2 diabetes and early-stage DR.

However, the results are inconsistent. Yilmaz et al. showed that serum APN concentrations are lower in patients with type 2 diabetes and that these concentrations are associated with the severity of DR [22]. Increased levels of IFN-γ and TNF-α in the vitreous have been found in patients with diabetes compared to non-diabetic patients [7]. By comparison, decreased levels of vitreous APN were found in patients with diabetes compared to those in non-diabetic patients [7]. One study also showed a discrepancy between serum and AH APN concentrations. Mao et al. [5] reported that AH APN concentration was increased in patients with PDR, but serum APN was not. Thus, they suggested that the blood-retinal barrier plays a key role in maintaining intraocular APN homeostasis.

To address this issue, we prospectively collected serum and AH samples on the same day. To reduce bias, patients were matched for both age and BMI. However, as shown in Table 1, we could not control for some baseline characteristics, such as the presence of HTN, HbA1c, statin use, creatinine, and AST. This variability seems to be inevitable because the DR grade may be related to the variables. To reduce the effects of other variables, we conducted a multivariate analysis. The relationship between serum and AH samples differed depending on cytokine levels. The log APN, log VEGF, log ESAM, IL-2, IL-6 and log leptin showed significant correlations between the two samples (p<0.001, R = 0.512; p = 0.001, R = 0.287; p<0.001, R = 0.321; p = 0.032, R = 0.186; p = 0.011, R = -0.219; and p = 0.003, R = 0.256; respectively). The cytokines that showed significant correlation had positive correlations, except for IL-6, which showed a significant negative correlation. However, ICAM-1, VCAM-1, IL-4, IL-5, IL-8, IL-10, and PTX3 did not show any significant differences between serum and AH samples (all P >0.05). This might be due to differences in the size, charge, and shape of the cytokines when penetrating the brood retinal barrier. Our team is currently conducting follow-up studies to evaluate this hypothesis.

Interestingly, as described above, APN (p<0.001, R = 0.512) showed a better correlation between serum and AH than VEGF (p = 0.001, R = 0.287), even though AH APN seemed as a better biomarker for estimating DR development and progression than serum APN (Table 4, Figs 1 and 3). Serum testing is more convenient and useful in many clinical situations when considering the amount of sample and the extent of invasiveness compared to extracting aqueous samples from the anterior chamber. Thus, knowing the extent of the correlation between serum and AH is very important when selecting a clinical biomarker. In addition, as VEGF regulation is a mainstay in the diagnosis and treatment of DR patients, good correlation with intraocular VEGF, including other cytokines, might indicate a role for APN in both diagnosis and treatment of DR development and progression.

This study has several strengths. First, we included consecutive patients who were carefully selected according to the criteria suggested by skilled specialists (HSY and CYJ), who were blinded to patient clinical information during DR grading. Only one eye per patient was selected randomly to reduce the possibility of bias. In addition, the present prospective study focused on the effect of both serum and intraocular APN concentrations on DR development to reduce the potential effects of various other cytokines in type 2 DM patients with and without DR. Despite these strengths, this study also had certain limitations. The sample size was relatively small, despite the study being based on a large population, so the subgroup analysis in the DR group did not have good statistical power for finding strongly positive factors. Moreover, although we compared age- and BMI-matched groups, the use of statins and the prevalence of HTN and HDL levels, which are believed to affect APN levels, could not be matched as described above. In addition, the relationship among various cytokines is not fully understood yet. For example, log APN in AH showed a significant negative correlation with some inflammatory cytokines such as log ICAM-1, lg PTX3, angiopoietin, and CRP. Ouch et al. reported that APN is a cardioprotective cytokine that inhibits IL-6, TNF-α, and Hs-CRP expressions, down-regulating monocytes chemoattractant protein-1 (MCP-1) [33]. Until now, we have not been able to understand this relation to the eyes. We can assume that acute localized inflammatory conditions (increased CRP or other inflammation cytokines) can happen in lowered systemic APN states with similar mechanism as seen by Ouch et al. We have tried to accumulate more evidence, such as CRP/APN ratio, suggested in cardiology research; however, this relationship should be studied more in future [34].

In conclusion, our study suggested that serum and AH APN levels were positively associated with DR development and progression. However, though AH APN showed better correlation than serum APN, clinically AH APN is not a convenient method to analyze APN due to the invasive nature of the procedure for such a small amount of the sample. Fortunately, serum APN showed a good correlation with the concentrations of various intraocular cytokines such as APN, VEGF, ICAM-1, leptin, PTX3, PDGF, angiopoietin, CRP, IL-5, and IL-10 in AH. Thus, serum APN might be used as a better predictive biomarker clinically for intraocular inflammation associated with DR development and progression than AH APN, AH VEGF, and serum VEGF. Future studies should validate the role of serum and AH APN and its mechanism of action in DR patients.

## Supporting information

S1 Data (XLSX)
